# Potentially burdensome care at the end-of-life for people who had a death from cancer: A retrospective population-level cohort study

**DOI:** 10.23889/ijpds.v9i5.2482

**Published:** 2024-09-18

**Authors:** Richard Booth, Cheryl Forchuk, Monidipa Dasgupta, Salimah Shariff

**Affiliations:** 1 ICES Western; 2 Arthur Labatt Family School of Nursing, Western University; 3 ICES; 4 Lawson Health Research Institute; 5 Division of Geriatric Medicine, Department of Medicine, Schulich School of Medicine and Dentistry, Western University

## Background

Cognitive decline in people experiencing homelessness (PEH) is an increasingly recognized issue. We compared the prevalence of dementia among PEH to housed individuals in the general population and those living in low-income neighborhoods. 

## Approach

A population-based cross-sectional comparative analysis using linked healthcare administrative data from Ontario, Canada. We included individuals ≥45 years on January 1, 2019 who visited hospital-based ambulatory care (eg, emergency department), were hospitalized, or visited a Community Health Centre in 2019; and identified PEH if they had one or more healthcare records with an indication of homelessness or unstable housing. Prevalence of dementia was ascertained using a validated case definition for Alzheimer’s disease and related dementia. Poisson models were used to generate estimates of prevalence.

## Findings

12,863 PEH, 475,544 low-income comparators, and 2,273,068 general population comparators were assembled. Among these groups, dementia prevalence was 68.7, 62.6, and 51.0 per 1,000, respectively. Descriptively, prevalence ratios between PEH and the comparator groups were highest within the ages of 55-64 and 65-74 in both sexes, ranging from 2.98 to 5.00. After adjusting for age, sex, geographical location of residence, and health conditions associated with dementia, prevalence of dementia among PEH was found to be 1.71 (95% CI: 1.60–1.82) and 1.90 (1.79–2.03) times greater than the low-income and general population comparators, respectively.

## Interpretation

PEH experience high burden of dementia compared to housed populations in Ontario. Findings suggest that PEH may experience dementia at younger ages and may benefit from the development of proactive screening and housing interventions.

